# Insulin Treatment Attenuates Small Nerve Fiber Damage in Rat Model of Type 2 Diabetes

**DOI:** 10.1155/2020/9626398

**Published:** 2020-08-02

**Authors:** Laura J. Andreasen, Rikke K. Kirk, Christian Fledelius, Mark A. Yorek, Jens Lykkesfeldt, Thorbjorn Akerstrom

**Affiliations:** ^1^Department of Veterinary and Animal Sciences, Faculty of Health and Medical Sciences, University of Copenhagen, 1870 Frederiksberg, Denmark; ^2^Global Drug Discovery, Novo Nordisk A/S, 2760 Måløv, Denmark; ^3^Department of Veterans Affairs Iowa City Health Care System, Iowa City, IA 52246, USA

## Abstract

**Introduction:**

Current clinical guidelines for management of diabetic peripheral neuropathy (DPN) emphasize good glycemic control. However, this has limited effect on prevention of DPN in type 2 diabetic (T2D) patients. This study investigates the effect of insulin treatment on development of DPN in a rat model of T2D to assess the underlying causes leading to DPN.

**Methods:**

Twelve-week-old male Sprague-Dawley rats were allocated to a normal chow diet or a 45% kcal high-fat diet. After eight weeks, the high-fat fed animals received a mild dose of streptozotocin to induce hyperglycemia. Four weeks after diabetes induction, the diabetic animals were allocated into three treatment groups receiving either no insulin or insulin-releasing implants in a high or low dose. During the 12-week treatment period, blood glucose and body weight were monitored weekly, whereas Hargreaves' test was performed four, eight, and 12 weeks after treatment initiation. At study termination, several blood parameters, body composition, and neuropathy endpoints were assessed.

**Results:**

Insulin treatment lowered blood glucose in a dose-dependent manner. In addition, both doses of insulin lowered lipids and increased body fat percentage. High-dose insulin treatment attenuated small nerve fiber damage assessed by Hargreaves' test and intraepidermal nerve fiber density compared to untreated diabetes and low-dose insulin; however, neuropathy was not completely prevented by tight glycemic control. Linear regression analysis revealed that glycemic status, circulating lipids, and sciatic nerve sorbitol level were all negatively associated with the small nerve fiber damage observed.

**Conclusion:**

In summary, our data suggest that high-dose insulin treatment attenuates small nerve fiber damage. Furthermore, data also indicate that both poor glycemic control and dyslipidemia are associated with disease progression. Consequently, this rat model of T2D seems to fit well with progression of DPN in humans and could be a relevant preclinical model to use in relation to research investigating treatment opportunities for DPN.

## 1. Introduction

Diabetic peripheral neuropathy (DPN) is the most common complication associated with type 2 diabetes (T2D) [1, 2] and affects up to 50% of the T2D population [3, 4]. Currently there is no treatment besides emphasizing good glycemic control. In type 1 diabetic patients, enhanced glycemic control is associated with a 78% relative risk reduction of developing DPN [4]. However, in T2D patients the incidence of DPN is not as dramatically reduced by tight glucose control, and enhanced glycemic control is only associated with a 5-9% relative risk reduction [4]. This indicates that hyperglycemia is less important to the development of DPN in T2D patients and that other mechanisms may be involved in disease progression. Studies have shown that the prevalence of DPN increases with obesity, dyslipidemia, hypertension, and hyperglycemia [5–7]. However, it is still unclear which of these components contribute to the onset and progression of DPN.

New therapies are needed to lower the incidence of DPN in T2D patients. To accelerate the development of these therapies, animal models with high predictive validity are needed. The high-fat-fed/low-dose streptozotocin-treated (STZ) rat has shown to be a good preclinical model of late-stage T2D exacerbating the metabolic disturbances such as dyslipidemia and insulin resistance observed in high-fat diet models [8, 9] but also adding hyperglycemia and low endogenous insulin levels to the disease model [10, 11]. Furthermore, high-fat-fed/low-dose STZ-treated rats also develop nerve damage mimicking DPN in human patients with decreased intraepidermal nerve fiber density (IENFD), decreased nerve conductance velocity, and abnormal nociception [10, 11]. However, the effect of insulin treatment on development of DPN has not been investigated in this rat model of T2D. Besides lowering blood glucose, insulin treatment may also affect lipid metabolism and body composition [12], which are known risk factors of DPN [13]. By treating with insulin, the contribution of hyperglycemia versus other risk factors may be assessed.

The purpose of this study was to investigate the effect of metabolic and weight change induced by insulin treatment on DPN measures in a rat model of T2D to elucidate the importance of hyperglycemia as a driving factor of DPN versus other risk factors also affected by insulin treatment.

## 2. Materials and Methods

### 2.1. Animals and Study Design

Male Sprague-Dawley rats aged 12 weeks were purchased from Charles River Laboratories in Italy. During the first six weeks of the study, the animals were housed at the Charles River Laboratories in France and the high-fat diet feeding (described below) was initiated there. For the remainder of the study period, animals were housed in the animal facilities at Novo Nordisk A/S in Måløv, Denmark. Animals were housed in ScanTainers (Scanbur, Karlslunde, Denmark) in groups of two and in rare instances single housed. Animals were kept at a light/dark cycle of 12 h/12 h with free access to water and food. The study was approved by the Danish Animal Experimental Inspectorate, Ministry of Environment and Food, Denmark in accordance with European Union directive 2010/63/EU.

**Figure 1:**
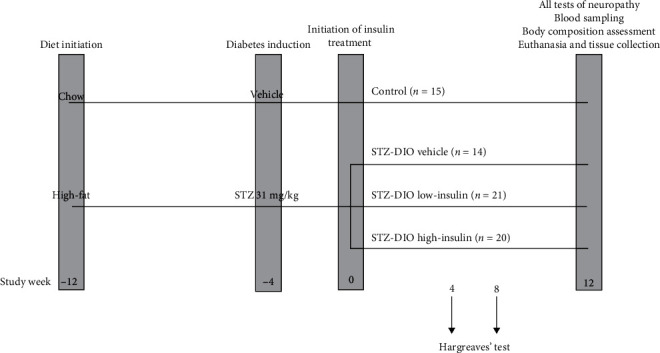
Overview of study design. For the diabetic groups, eight weeks of high-fat feeding were followed by a mild dose of streptozotocin (STZ, s.c., 31 mg/kg) to induce hyperglycemia. Animals had untreated diabetes for four weeks, and hereafter, treatment with vehicle or insulin-releasing implants were initiated and continued for 12 weeks. To monitor the insulin treatment, nonfasted morning blood glucose and body weight were measured weekly. Hargreaves' test to assess thermal induced pain was performed four, eight, and 12 weeks after treatment initiation. In addition to Hargreaves' test, motor nerve conductance velocity measurements, blood sampling, and body composition assessment were made at study termination followed by euthanasia and tissue collection. Control animals received standard rodent chow during the whole study and were treated with vehicle implants during the treatment period but otherwise handled and tested as the diabetic animals. STZ-DIO: streptozotocin-treated diet-induced obese.

Animals were allocated to either a 45% kcal high-fat diet (Research Diet, D12451, New Brunswick, NJ, USA) or standard rodent chow (Altromin International, Altromin 1324, Lage, Germany) based on initial body weight using the minimization method [14]. After eight weeks of diet, rats fed a high-fat diet were treated with a mild dose of STZ (s.c., 31 mg/kg in 0.1 M citric acid buffer at pH 4.5) to induce hyperglycemia, while animals fed chow were sham-injected with vehicle. Even though STZ is a toxic compound, it has been shown not to induce neurotoxicity in rodents and thereby interfere with the nerve endpoints [15]. Rats with a blood glucose of 13.8 mM or greater were considered diabetic, as described elsewhere [11]. Four weeks after diabetes induction, the streptozotocin-treated diet-induced obese (STZ-DIO) animals were allocated using minimization [14] based on body weight, body fat percentage, and HbA_1c_% to three treatment groups (STZ-DIO high-insulin, STZ-DIO low-insulin, and STZ-DIO vehicle) receiving either insulin-releasing or vehicle implants (LinShin Canada Inc., Linplant, Ontario, Canada). A 12-week treatment period was chosen not only because it would gain a stable treatment condition but also because this would result in 16 weeks of hyperglycemia which has resulted in substantial nerve damage in other studies using the same model [10, 11, 16]. Nonfasted morning blood glucose and body weight were monitored weekly during the whole treatment period. Furthermore, body composition was assessed by a quantitative magnetic resonance scan (Echo Medical Systems, EchoMRI™, Houston, TX, USA) pre- and posttreatment and fat mass quantified by the scan together with body weight was used to calculate body fat percentage. An overview of the study design can be seen in Figure 1.

### 2.2. Insulin and Vehicle Treatment

The insulin-releasing implants contained human insulin and were inserted s.c. in the neck by a trocar as instructed by the manufacturer while animals were under isoflurane anesthesia (3% isoflurane concentration with a flow rate of 0.7 L/min O_2_ and 0.3 L/min N_2_O). STZ-DIO high-insulin received four insulin-releasing implants per kg and STZ-DIO low-insulin received two insulin-releasing implants per kg, whereas STZ-DIO vehicle and control were allocated to two or four vehicle implants per kg. No differences in blood glucose between two and four vehicle implants per kg was observed in either STZ-DIO or control, and consequently, animals were pooled to one STZ-DIO vehicle group and one control group.

After approximately five weeks, new insulin-implants were inserted, but only a half-dose was given compared to the first insertion to avoid hypoglycemia. Hereafter, animals in STZ-DIO high-insulin were given additional insulin implants if glucose control was not tight enough (blood glucose > 13.8 mM).

### 2.3. Thermal Induced Pain

Hargreaves' test (Ugo Basile, The Plantar Test Instrument, Gemonio, Italy) was performed four, eight, and 12 weeks after treatment initiation to investigate thermal induced pain. Animals were acclimatized to the lab for 30 minutes before they were placed in a clear plexiglas box on top of the plantar test apparatus. Rats were acclimatized to the glass surface and surroundings for five minutes. After acclimatization, the mobile infrared heat source was positioned underneath the hind paw of the animal and turned on by the operator, starting a timer that stopped when the rat withdrew its paw. An automatic shut off was set to 30 sec to prevent tissue damage. The intensity of the infrared heat source was set at 55% of maximal, corresponding to a flux of energy of approximately 130 mW/cm^2^ through the plate. Four to five animals were tested at a time, and latency was measured six times for each animal. If the animal did not respond with a clear withdrawal of the paw, a new measurement was taken. Of the last five successful latency measurements, the three measurements with the closest numerical values from each animal were used to calculate mean latency. The operator was blinded to group allocation.

A familiarization to the Hargreaves' test was performed one week prior to the first test. Animals were acclimatized as described above, and latency was measured two to three times to familiarize the rat with the procedure.

### 2.4. Motor Nerve Conductance Velocity

**Figure 2:**
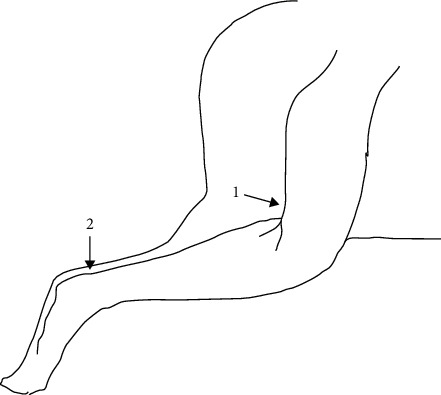
Illustration of the two sites where the sciatic-posterior tibial conducting system was stimulated during motor nerve conduction velocity measurements: (1) posterior to the knee joint before branching of the sciatic nerve and (2) at the Achilles tendon near the calcaneus.

After 12 weeks of treatment, motor nerve conductance velocity (MNCV) measurements were performed to assess large nerve fiber function. Animals were anaesthetized with isoflurane (3% isoflurane concentration with a flow rate of 0.7 L/min O_2_ and 0.3 L/min N_2_O). To avoid temperature loss, animals were placed on a heating pad (World Precision Instruments, ATC2000, Sarasota, FL, USA) and temperature was monitored with a rectal probe with a setpoint of 37°C. The left sciatic nerve was stimulated proximally at a 45° angle and approximately 0.5 cm behind the knee joint before nerve branching, also referred to as the sciatic notch by other authors [17], and distally at the Achilles tendon near the calcaneus (n. tibialis). An illustration of the stimulation sites can be seen on Figure 2. Stimulation consisted of single 0.1 ms supramaximal (8-10 V) pulses through a bipolar electrode (Natus Neuro, Dantec™ DCN Disposable Concentric Needle Electrode 25 mm × 30G, Middleton, WI, USA). The stimulus was recorded underneath the foot near the first interosseous muscle also with a bipolar electrode (Natus Neuro, Dantec™ DCN Disposable Concentric Needle Electrode 25 mm × 26G). A ground electrode (Technomed, Disposable Subdermal Single Needle Electrodes, Maastricht, The Netherlands) was placed s.c. in the flank to limit noise. The evoked potential was displayed on a digital storage oscilloscope (RS Components, RSDS1000 Series #123-6628, Corby, UK), and latency was measured from stimulus artifact to peak amplitude of the recorded evoked potential. The distance between the proximal and distal stimulation site was measured with a digital Vernier caliper (Mitotoyo, CD-15APX, Kawasaki, Japan). MNCV was calculated as the distance between the distal and proximal stimulation site divided by the latency difference between the proximal and distal site. The operator was blinded to group allocation.

### 2.5. Euthanasia and Tissue Collection

Immediately after MNCV measurements, animals were euthanized by cervical dislocation. The sciatic nerve of the right midthigh was dissected out, put into a cryogenic vial (Fisher Scientific International, 1.8 mL Nunc™ Cryogenic Tubes, MA, USA), and snap frozen in liquid nitrogen. The samples were stored at -80°C until further analysis. The right foot was removed and fixated in 10% neutral buffered formalin until further processing.

### 2.6. Intraepidermal Nerve Fiber Density in the Hind Paw

Immunohistochemistry was used to visualize small unmyelinated nerve fibers within the epidermis. The skin from underneath the fixated foot was trimmed with a scalpel. Skin samples were processed in a tissue processor (Leica Biosystems, ASP300S, Wetzlar, Germany), embedded in paraffin wax, and sections of 15 *μ*m thickness were cut on a microtome (Leica Biosystems, Leica RM2255) and mounted on a SuperFrost® Plus slide (Thermo Scientific, Waltham, MA, USA). Prior to staining, slides were put in a 37°C incubator for approximately 48 hours followed by 60 minutes at 60°C to prevent tissue from falling off the slides when stained. Immunohistochemical staining with an anti-PGP9.5 antibody in 1 : 333 dilution for 40 minutes at room temperature (Abcam, ab108986, Cambridge, UK) followed by treatment with a secondary antibody (Roche, DISCOVERY Anti-Rabbit HQ, Basal, Switzerland) for 16 minutes at room temperature was performed using a fully automated staining platform for one slide per animal (Roche, Ventana Discovery ULTRA). As controls, two slides with either known high or low PGP9.5 expression were used. At every run, both control slides were either stained with the primary antibody, stained but omitting the primary antibody, or stained with an IgG isotype control in the same concentration as the primary antibody (Cell Signaling Technology®, Rabbit (DA1E) mAb IgG XP® #3900, Danvers, MA, USA). After staining, slides were washed and mounted (Dako, Fluorescence Mounting Medium, Carpinteria, CA, USA). A Z-scan at 10x magnification with a depth direction of 8/8 *μ*m was performed to digitalize the stained slides (Olympus, Olympus V120, Tokyo, Japan). All immunoreactive profiles within the epidermis were counted in OlyVIA (Olympus, version 2.4) and normalized to epidermal length measured in ImageJ software (National Institutes of Health, Bethesda, MD, USA). The investigators counting the profiles were blinded to the sample identity.

### 2.7. Sorbitol and *myo*-Inositol Content in the Sciatic Nerve

Sciatic nerve tissue was processed and analyzed for sorbitol and *myo*-inositol content as previously described [18, 19].

### 2.8. Blood Analyses

Nonfasted morning sublingual blood samples were taken two days prior to euthanasia. Blood was EDTA-stabilized in Microvette® tubes (Sarstedt, 500 K3E, Nümbrecht, Germany) and kept on wet ice for no more than 30 minutes before centrifugation at 2000 × g for 10 minutes at 4°C. Before centrifugation, two 10 *μ*L samples of whole blood were collected in heparin-coated capillary tubes. One was mixed with 500 *μ*L Biosen buffer, and blood glucose was directly analyzed using a Biosen autoanalyzer (EKF Diagnostics, Biosen S_Line, Cardiff, UK) according to the manufacturer's instructions. The other was mixed with 500 *μ*L hemolyzing reagent and stored at -20°C until HbA_1c_ analysis was performed on a Cobas 6000 autoanalyzer (Roche, Cobas c501 module) according to the manufacturer's instruction. All plasma samples were stored at -20°C until analysis.

Human insulin and rat C-peptide plasma levels were analyzed using in-house-developed luminescent oxygen channeling immunoassays as described previously [20]. Coupling of antibodies to beads, biotinylation of antibodies, and the luminescent oxygen channeling immunoassay procedure were performed as previously described [21]. Calibrators and quality control samples were produced in the same matrix as the study samples. Assay precision was assessed and shown to be lower than 20% for all the tested samples. For the quantification of human insulin levels in rat plasma, the assay used (anti-human insulin) HUI018 monoclonal antibody-conjugated acceptor beads together with biotinylated OXI-005 monoclonal antibody (raised against human insulin) and generic streptavidin-coated donor beads. Cross-reactivity to endogenous rat insulin was lower than 0.1%. For the quantification of rat C-peptide levels in plasma, the assay used anti-rat C-peptide HyTest CC27 monoclonal antibody- (HyTest Ltd., Turku, Finland) conjugated acceptor beads and biotinylated HyTest CII-29 monoclonal antibody- (HyTest Ltd.) and generic streptavidin-coated donor beads. Plasma triglyceride (TG) and total cholesterol (TC) concentrations were analyzed using the Cobas 6000 autoanalyzer (Roche) according to the manufacturer's instructions.

### 2.9. Statistical Analyses

Group differences were evaluated using a one-way analysis of variance (ANOVA) with group as the class variable. If an overall difference was found, a post hoc test with the Tukey-Kramer adjusted *P* values for multiple testing was performed. Logarithmic transformation of data was used when considered appropriate, to achieve normal distribution and variance homogeneity of residuals. If variance homogeneity of residuals could not be obtained by logarithmic transformation, a nonparametric Kruskal-Wallis test with the Dunn multiple comparison test was performed.

In addition, linear regression analysis was used to test for associations between metabolic parameters (HbA_1c_%, TG, TC, body fat percentage, and sciatic nerve sorbitol content) and markers of peripheral nerve damage (latency from Hargreaves' test, IENFD, and MNCV). Logarithmic transformation of the independent variable was used when considered appropriate, to achieve normal distribution and variance homogeneity of residuals.

Furthermore, an analysis of covariance (ANCOVA) was performed to investigate if the abovementioned metabolic parameters could explain differences in nerve damage between the treatment groups. The ANCOVA was performed with group as class variable, latency from Hargreaves' test or IENFD as response variable, and the metabolic parameters significant in the linear regression as covariates. All parameters were included in a model with stepwise backwards reduction until only significant findings were left. When appropriate, data were transformed to fit model assumptions. MNCV was not run as a response variable in the ANCOVA because no difference between groups was found in the ANOVA.

Data are presented as mean and standard deviation (SD). Statistical analyses were made using statistical software (SAS Institute Inc., SAS Enterprise Guide® 7.1, Cary, NC, USA). Graphical illustrations were made using GraphPad Prism® (GraphPad Software Inc., 8.0 for Windows, San Diego, CA, USA). A value of *P* < 0.05 was considered statistically significant.

## 3. Results

### 3.1. Metabolic Characteristics and Body Composition

**Figure 3:**
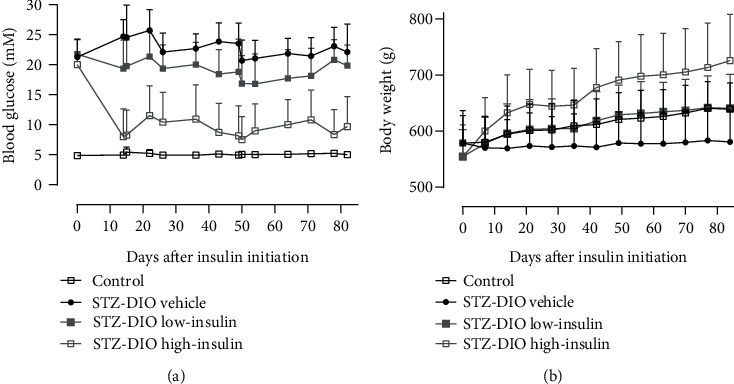
Effect of diabetes and insulin treatment on (a) nonfasted morning blood glucose levels and (b) body weight. Data are presented as mean and SD.

Results of glycemic control, circulating lipids, and body composition before treatment initiation and at study termination are presented in Table 1. Before treatment initiation, HbA_1c_%, TG, and TC were higher in the three diabetic groups compared to the nondiabetic animals, whereas C-peptide level was lower. Average blood glucose levels, calculated from the weekly blood glucose measurement, were significantly different between all groups, and different degrees of glycemic control were thereby achieved, as depicted on Figure 3(a), where all blood glucose measurements during the treatment period can be seen. At study termination, HbA_1c_% was significantly higher in the three diabetic groups compared to the control. Furthermore, animals in STZ-DIO high-insulin had significantly lower HbA_1c_% compared to the two other diabetic groups. Plasma C-peptide level was significantly lower in the diabetic groups compared to the control confirming *β*-cell damage by STZ. Human insulin levels in plasma corresponded to the dose of insulin implants given. Plasma TG and TC were significantly increased in the diabetic groups compared to the control. Both doses of insulin lowered plasma TG and TC levels compared to STZ-DIO vehicle not receiving insulin, but no difference in plasma lipids was observed between the two insulin doses.

Before treatment initiation, body weight and body fat percentage were similar between all four groups. After 12 weeks of insulin treatment, diabetic animals receiving high- and low-dose insulin had a significantly higher body weight and body fat percentage compared to untreated diabetic animals. Furthermore, high-dose insulin increased body weight and body fat percentage significantly more than low-dose insulin. The body weight data at study termination correspond to Figure 3(b), where it is shown that the rate of body weight gain is highest in the high-insulin group, lowest in the diabetic group not receiving insulin, and in between the two other groups for the low-insulin group.

### 3.2. Neuropathy Endpoints

**Figure 4:**
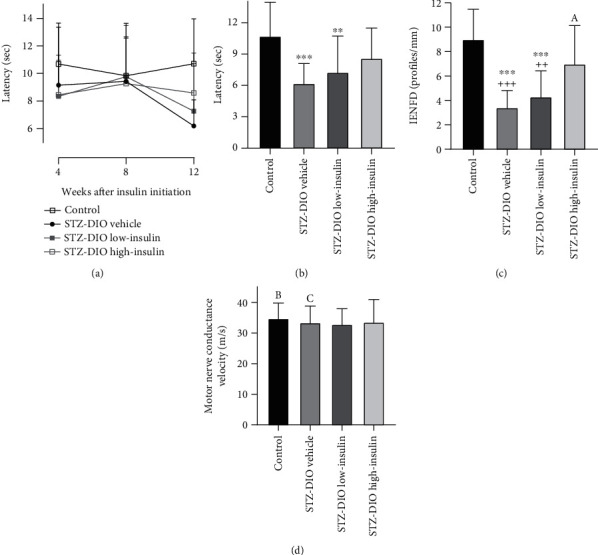
Effect of diabetes and insulin treatment on peripheral neuropathy. (a) Development of thermal induced pain assessed by Hargreaves' test during the treatment period, and (b) thermal induced pain assessed by Hargreaves' test, (c) intraepidermal nerve fiber density (IENFD), and (d) motor nerve conductance velocity at study termination. Data are presented as mean and SD. ^∗∗^*P* < 0.01 and ^∗∗∗^*P* < 0.001, significantly different from control. ^++^*P* < 0.01 and ^+++^*P* < 0.001, significantly different from STZ-DIO high-insulin. Control, *n* = 15; STZ-DIO vehicle, *n* = 14; STZ-DIO low-insulin, *n* = 21; STZ-DIO high-insulin, *n* = 20 except in cases where indicated otherwise, A: *n* = 19; B: *n* = 14; C: *n* = 13.

**Figure 5:**
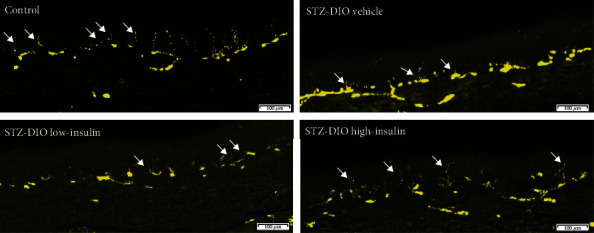
Example of intraepidermal nerve fibers in each of the four groups. White arrows point out intraepidermal nerve fibers (right corner bar is 100 *μ*m).

Four and eight weeks after insulin treatment initiation, no difference in thermal induced pain was found between groups (Figure 4(a)). However, 16 weeks after diabetes induction and 12 weeks after treatment initiation, thermal hyperalgesia as evident by a decrease in latency was observed in animals receiving low-insulin or no insulin compared to control. Treatment with a high dose of insulin resulted in latencies that were numerically in-between the control and STZ-DIO vehicle but not significantly different from any of the other groups (Figure 4(b); control: 10.7 ± 3.2; STZ-DIO vehicle: 6.2 ± 1.9; STZ-DIO low-insulin: 7.3 ± 3.5; STZ-DIO high-insulin: 8.6 ± 2.9 seconds). Loss of small nerve fibers in STZ-DIO vehicle and STZ-DIO low-insulin was evident by the significantly lower IENFD in these groups compared to both control and STZ-DIO high-insulin. A tendency towards lower IENFD was observed in STZ-DIO high-insulin (*P* = 0.09) compared to control (Figure 4(c); control: 9.0 ± 2.5; STZ-DIO vehicle: 3.4 ± 1.4; STZ-DIO low-insulin: 4.3 ± 2.1; STZ-DIO high-insulin: 7.0 ± 3.1 profiles/mm). On Figure 5, a representative example of IENFD in each group can be seen. MNCV was not different between groups (*P* = 0.8; Figure 4(d); control: 34.7 ± 5.1; STZ-DIO vehicle: 33.4 ± 5.5; STZ-DIO low-insulin: 32.8 ± 5.1; STZ-DIO high-insulin: 33.6 ± 7.4 m/s), suggesting no large nerve fiber damage after 16 weeks of hyperglycemia.

### 3.3. Sciatic Nerve Sorbitol and *myo*-Inositol Content

**Figure 6:**
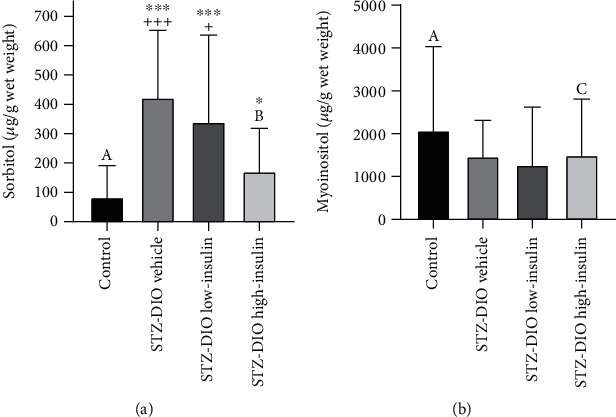
Effect of diabetes and insulin treatment on sciatic nerve (a) sorbitol and (b) *myo*-inositol contents. Data are presented as mean and SD. ^∗^*P* < 0.05 and ^∗∗∗^*P* < 0.001, significantly different from control. ^+^*P* < 0.05 and ^+++^*P* < 0.01, significantly different from STZ-DIO high-insulin. Control, *n* = 15; STZ-DIO vehicle, *n* = 14; STZ-DIO low-insulin, *n* = 21; STZ-DIO high-insulin, *n* = 20 except in cases where indicated otherwise, A: *n* = 14; B: *n* = 18; C: *n* = 19.

Diabetes increased sorbitol content in the sciatic nerve with all three diabetic groups having a higher level of sorbitol compared to control. However, treatment with a high dose of insulin decreased sorbitol content in the sciatic nerve compared to a low dose of insulin and no insulin (Figure 6(a)). No difference between groups in sciatic nerve *myo*-inositol content was observed (*P* = 0.2, Figure 6(b)).

### 3.4. Association between Metabolic Parameters and Neuropathy Endpoints

**Figure 7:**
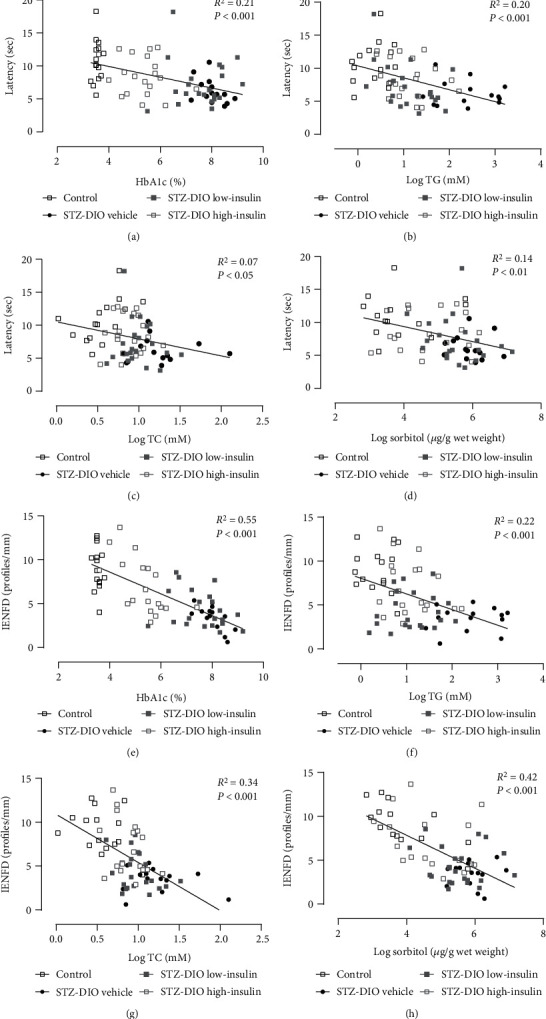
Associations between metabolic parameters and markers of peripheral nerve damage from simple linear regression analysis. Only significant findings are illustrated. Logarithmic transformation of the independent variable was performed to obtain normal distribution and variance homogeneity of residuals. IENFD = intraepidermal nerve fiber density, TG = triglyceride, and TC = total cholesterol.

Simple linear regression analysis revealed a negative association between small nerve fiber damage and the following metabolic parameters: HbA_1c_%, sciatic nerve sorbitol content, and circulating TG and TC (Figure 7); this indicates that small nerve fiber damage progressed with increasing glucose and lipid level.

As insulin (directly or indirectly) is known to affect the abovementioned metabolic parameters, an ANCOVA was made to elucidate if the positive effect of insulin on small nerve fiber damage could be explained by its effects on circulating glucose and lipids. None of the covariates significantly explained the differences found between treatment groups in Hargreaves' test. However, a tendency towards plasma TG level being associated with changes in thermal induced pain independent of treatment group was found (Table 2), as group was nonsignificant in the analysis. HbA_1c_% and plasma TC were significant in the ANCOVA with IENFD as response variable and treatment group was nonsignificant in the analysis (Table 2). The influence of HbA_1c_% and plasma TC status on IENFD was therefore stronger than treatment *per se*, suggesting that IENFD may be better explained by HbA_1c_% and plasma TC level than by treatment group.

## 4. Discussion

In this study, insulin treatment was used to create different levels of glycemic control in a rat model of T2D. Besides lowering blood glucose in a dose-dependent manner, both doses of insulin decreased circulating lipids and increased body fat percentage. The high-dose insulin treatment attenuated small nerve fiber damage assessed by Hargreaves' test and IENFD, but the same protective effect was not observed for low-dose insulin treatment. Interestingly, we found that loss of intraepidermal nerve fibers and thermal hyperalgesia were associated with poor glycemic control and dyslipidemia.

That abnormalities in glucose alone cannot explain the peripheral nerve damage in the present study is in line with data from large randomized controlled trials, where DPN was not prevented solely by intensive glycemic control [3, 22]. However, more recent studies have shown that improvements in neuropathy outcomes are possible by normalizing HbA_1c_ in T2D patients [23–25]. This suggests that glycemic control might play a larger role in disease progression in T2D patients, but considerably tighter glycemic control than recommended is needed to obtain improvements in neuropathy. Hyperglycemia has been linked to the development of DPN through several pathways. In the hyperglycemic state, excessive glucose can cause an overload of metabolites to the mitochondria in the nerves, which may lead to increased generation of reactive oxygen species and mitochondrial dysfunction [26, 27]. Moreover, glucose may enter the alternative polyol pathway when the oxidative glucose metabolism of the mitochondria is overloaded [26, 27]. The polyol pathway reduces glucose to sorbitol and fructose, which also increases oxidative stress formation and inflammatory signaling [26, 27]. In the present study, a lower content of sorbitol was observed in the sciatic nerve of animals receiving a high-dose insulin treatment compared to the diabetic animals receiving a low dose or no insulin treatment (Figure 6(a)). This suggests that flux through these alternate pathways was decreased by lowering circulating glucose levels, which may subsequently have resulted in less small nerve fiber damage as observed in the high-insulin group. In the present study, sciatic nerve sorbitol content was also negatively associated with small nerve fiber damage (Figures 7(d) and 7(h)), underlining the possible link between decreased flux through the polyol pathway and nerve damage. Furthermore, increased HbA_1c_% was strongly associated with a decline in IENFD and thermal hyperalgesia (Figures 7(a) and 7(e)) also independent of insulin dose (Table 2), indicating that in this rat model of T2D, hyperglycemia contributes to the development of peripheral nerve fiber damage and that glycemic control to some extent may attenuate these damages, as also observed in more recent human studies [23, 24].

An association between dyslipidemia and DPN has been shown in several studies with diabetic patients [6, 28–30]. In one of the studies, the effect of acetyl-L-carnitine was investigated, and subsequently, data were reanalyzed with the purpose of studying the mechanisms underlying DPN. They showed that both HbA_1c_% and TG levels correlated with progression of DPN. However, in a secondary analysis taking baseline nerve damage into account, only TG level was associated with rapid progression of DPN. Furthermore, a machine-learning paradigm was used to test which factors could predict DPN progression. Here TG, TC, and clinical symptom score had the most influence on DPN progression [29]. Based on these observations, it could be argued that dyslipidemia may be more important to progression of DPN than hyperglycemia in T2D patients. This conclusion would be in line with the finding that nerve damage can be observed in the prediabetic state before overt hyperglycemia [31, 32]. Conversely, the present study may suggest that glucose lowering is more important than lowering lipids because plasma TG and TC were decreased to a similar level in STZ-DIO high-insulin and STZ-DIO low-insulin (Table 1), as insulin inhibits adipose tissue lipolysis, stimulates uptake of fatty acids, and modulates the liver's apoprotein production [33] and thereby lowering circulating lipids. In contrast, attenuation of small nerve fiber damage was only observed in the high-dose insulin-treated animals (Figures 4(b) and 4(c)), where more tight glucose control was obtained. However, linear regression analysis revealed that increased circulating levels of both glucose and lipids were associated with small nerve fiber damage (Figure 7), indicating that both glucose and lipids are involved in the pathogenesis of DPN. Similarly, other studies have reported an association between the development of DPN and both glucose and lipid metabolism [6, 34]. Furthermore, studies investigating transcriptional profiling of *db*/*db* mice and human T2D patients with DPN have found that genes related to both glucose and lipid metabolism are changed in the nerves [35, 36], indicating an involvement of both pathways in disease progression and emphasizing the complexity of the disease.

Finally, it has also been suggested that insulin *per se* may have a neurotropic effect because low insulin levels and reduced neural insulin signaling have been associated with signs of DPN in normoglycemic rodents [37]. It has been demonstrated that local application (eye and intraplantar) of insulin in insulinopenic rodents is able to prevent the nerve depletion in cornea and epidermis without lowering blood glucose levels [38, 39], supporting the notion that insulin has a direct effect on nerves in addition to glucose lowering. In the present study, it is difficult to separate the neurotropic effect of insulin from its glucose lowering effects because a dose-dependent lowering of blood glucose was seen with insulin treatment (Table 1 and Figure 3(a)). However, animals in the low-insulin group were severely hyperglycemic despite insulin treatment and had the same level of small nerve fiber damage as the untreated diabetic animals (Figures 4(b) and 4(c)). In the present model of T2D, s.c. low-dose insulin treatment did therefore not reverse signs of DPN without improving glycemic control significantly as observed in other studies [37, 40]. The neurotropic effect of insulin is mostly supported by animal studies of insulin-deficient rodents, and even though the animals in the current study have been treated with STZ, some endogenous insulin production remains and the loss of neurotropic signaling may therefore not be as pronounced as in insulin-deficient rodents.

In this study, diabetes did not cause large nerve fiber damage when assessed by MNCV (Figure 4(d)). That 16 weeks of untreated diabetes was not enough to cause a decline in MNCV was surprising because decreased MNCV has been observed in other studies of similar length using similar rat models of T2D [10, 11, 16]. Increased sorbitol and decreased *myo*-inositol content in the sciatic nerve have been associated with decreased MNCV in experimental type 1 diabetes [17]. Here, we only observed increased sciatic nerve sorbitol content and no significant change in *myo*-inositol in the diabetic animals (Figures 6(a) and 6(b)). It has been suggested that hyperglycemia and sorbitol accumulation are driving the *myo*-inositol depletion and hence the decreased Na^+^-K^+^-ATPase activity leading to decreased nerve conductance [41]. Because no change in sciatic nerve *myo*-inositol content was observed, one may speculate that diabetes duration was not long enough to cause slowing of MNCV. When investigating the progression of DPN, small unmyelinated nerve fibers are typically affected first, and impairment of large nerve fiber function occurs later in disease progression [42–44]. It took 16 weeks of diabetes before differences between groups in thermal induced pain could be detected (Figure 4(a)), supporting that the diabetic animals in the present study may be in the early stages of DPN where only small nerve fiber damage is observed. This could have been elucidated if nerve deficits had been investigated prior to insulin treatment initiation. Furthermore, this lack of pretreatment tests makes it difficult to completely determine if treatment slowed progression or reversed the nerve damages observed.

Because slowing of MNCV was not observed in this study, there might be a difference in the neuropathic phenotype between the animals of the present study and the animals used in other studies [10, 11, 16]. This is further supported by the different effects of long-term diabetes on thermal induced pain (hypoalgesia vs. hyperalgesia) between the present study (Figures 4(a) and 4(b)) and other studies [10, 11, 16]. Vendor-derived differences in rats are a described phenomenon in other studies in respect to different behavioral, metabolic, and pain-related parameters [45–47] and might explain the different observations. That slowing of MNCV takes a longer time to develop in the present neuropathic phenotype may increase the validity of the model, since development of MNCV deficits also progress slowly in human T2D patients [42, 44]. However, studies investigating if a longer period of hyperglycemia would lead to large nerve fiber damage in the present neuropathic phenotype need to be performed to clarify this.

## 5. Conclusion and Perspectives

In summary, we show that small nerve fiber damage is attenuated by high-dose insulin treatment in a rat model of T2D. Furthermore, our data suggest that both poor glycemic control and dyslipidemia are associated with small nerve fiber damage. This rat model of T2D therefore resembles the complex pathogenesis in human T2D patients where both abnormal glucose and lipid metabolism seem to be important for the onset and progression of DPN. However, in this study, 16 weeks of diabetes was not sufficient to cause large nerve fiber damage, indicating that the diabetic animals were in the early stages of DPN.

Future studies should focus on lowering of both glucose and lipids to investigate if this can stop the progression of DPN.

## Figures and Tables

**Table 1:** Glycemic control, circulating lipids, and body composition before treatment initiation and at study termination.

	Control *n* = 15	STZ-DIO vehicle *n* = 14	STZ-DIO low-insulin *n* = 21	STZ-DIO high-insulin *n* = 20	Overall *P* value (ANOVA)
*Glycemic control and circulating lipids*					
Average blood glucose (mM)	5.1 ± 0.2^A^	22.9 ± 1.8^B^	19.0 ± 3.3^C^	9.3 ± 3.3^D^	<0.001
HbA_1c_ (%)					
Pretreatment	3.5 ± 0.2^A^	7.3 ± 0.5^B^	7.3 ± 0.5^B^	7.2 ± 0.4^B^	<0.001
Posttreatment	3.5 ± 0.1^A^	8.0 ± 0.9^B^	7.6 ± 0.9^B^	5.3 ± 0.5^C^	<0.001
C-peptide (pM)					
Pretreatment	589 ± 156^A^	92 ± 37^B^	103 ± 75^B^	99 ± 52^B^	<0.001
Posttreatment	809 ± 294^A^	95 ± 27^B^	126 ± 90^B^	140 ± 109^B^	<0.001
Human insulin (pM)					
Posttreatment^∗^	0 ± 0^A^	0 ± 0^A^	61 ± 29^B^	289 ± 219^C^	<0.001
Triglyceride (mM)					
Pretreatment	1.37 ± 0.41^A^	12.25 ± 9.79^B^	11.25 ± 6.62^B^	10.31 ± 5.97^B^	<0.001
Posttreatment	1.52 ± 0.51^A^	12.95 ± 7.43^B^	3.55 ± 1.88^C^	3.46 ± 2.02^C^	<0.001
Total cholesterol (mM)					
Pretreatment	1.74 ± 0.45^A^	2.78 ± 0.89^B^	3.20 ± 1.26^B^	2.83 ± 0.81^B^	<0.001
Posttreatment	1.78 ± 0.46^A^	3.65 ± 1.57^B^	2.76 ± 0.63^C^	2.45 ± 0.47^C^	<0.001
*Body composition*					
Body weight (g)					
Pretreatment	578 ± 49^A^	578 ± 58^A^	555 ± 48^A^	554 ± 57^A^	0.3
Posttreatment	639 ± 47^AB^	581 ± 56^A^	641 ± 60^B^	726 ± 60^C^	<0.001
Body fat percentage (%)					
Pretreatment	7.9 ± 2.7^A^	8.4 ± 2.5^A^	8.3 ± 2.9^A^	7.7 ± 2.1^A^	0.8
Posttreatment	10.5 ± 3.0^AB^	7.4 ± 2.1^A^	13.8 ± 4.1^B^	19.2 ± 4.7^C^	<0.001

Data are presented as mean and SD. Group differences tested by one-way ANOVA followed by a post hoc test with the Tukey-Kramer adjusted *P* values. Values sharing the same superscript letter are not statistically different. ^∗^Nonparametric analysis.

**Table 2:** Influence of different metabolic parameters on nerve damage using analysis of covariance.

Response variable	Explanatory variable and covariates	Parameter estimate	Standard error	*P* value
(a) Thermal induced pain	TG^∗^	-1.3	0.7	0.07
	Treatment group	—		0.18

(b) IENFD	HbA_1c_%	-1.4	0.4	<0.001
	TC	-0.7	0.3	0.04
	Treatment group	—		0.7

The response variables (a) thermal induced pain and (b) intraepidermal nerve fiber density (IENFD) were evaluated in an analysis of covariance using stepwise backwards reduction leaving only significant covariates with group as class variable and HbA_1c_%, sciatic nerve sorbitol content, triglyceride (TG), and total cholesterol (TC) as covariates to investigate the influence of the covariates on nerve damage. Only significant findings are shown. ^∗^Log transformed.

## Data Availability

The data used to support the findings of this study are available from the corresponding author upon request.
